# Vulvar dermatoses and depression: A systematic review of vulvar lichen sclerosus, lichen planus, and lichen simplex chronicus

**DOI:** 10.1016/j.jdin.2023.10.009

**Published:** 2023-12-01

**Authors:** Feben Messele, Kathryn Hinchee-Rodriguez, Christina N. Kraus

**Affiliations:** a School of Medicine, University of California Irvine, Irvine, California; b Department of Dermatology, University of California Irvine, Irvine, California

**Keywords:** depression, depressive symptoms, lichen planus, lichen sclerosus, vulvar disease

*To the Editor:* Lichen sclerosus (LS), lichen planus (LP), and lichen simplex chronicus are 3 of the most common vulvar inflammatory dermatoses (VID), and significantly affect patients’ quality of life and well-being.^1^ Depression is reported in up to 30% of patients with dermatologic disorders, which affects compliance and treatment outcomes.^2^ However, there is limited data on the prevalence and measures to assess depression in patients with VID. Here, we performed a systematic review on depression in VID to evaluate prevalence and identify instruments utilized to assess depression.

We conducted searches of PubMed, Cochrane, Scopus, and Web of Science in January 2022. Database searches included articles published between 1994 and 2021 in the English language using MeSH search terms Vulvar Diseases, Pruritus Vulvae, Atrophic Vaginitis, Depression, OR Depressive Disorders. Initial searches yielded 236 unique studies (Fig 1). Inclusion criteria: patients any age with 1 or more VID (LS, LP, lichen simplex chronicus), screened for or diagnosed with depression/depressive symptoms. Exclusion criteria: nonhuman studies, vulvodynia, vulvar pain syndromes without skin disease, infectious disease, vulvar intraepithelial neoplasia, and vulvar neoplasms.

**Fig 1:**
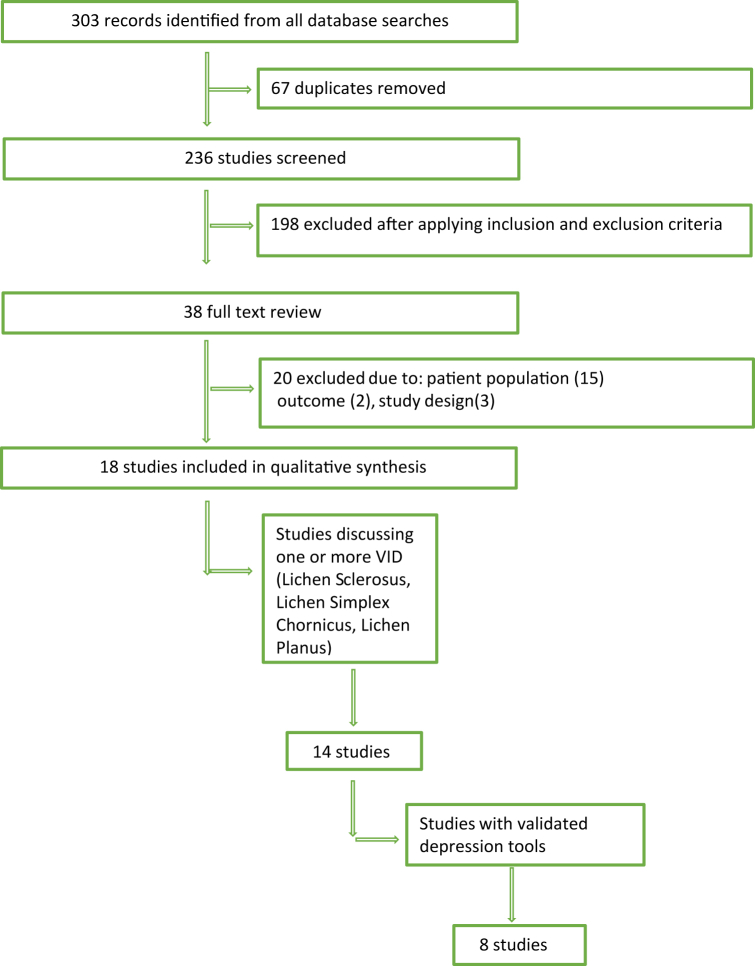
Preferred Reporting Items for Systematic Reviews and Meta-Analyses flow diagram. *VID*, Vulvar inflammatory dermatoses.

Ultimately, 14 studies of 1530 patients were included (Table I). Of these, 8 used validated depression scales, consisting of 656 patients (Table I).^3^ Validated instruments included: Hospital Anxiety and Depression Scale (3 studies), Beck’s Depression Inventory (1), Patient Health Questionnaire (1), Hamilton Depression Scale (1), and Center for Epidemiologic Studies Depression Scale (2).

**Table I:** Studies on vulvar inflammatory dermatoses and depression or depressive symptoms: demographics, vulvar diagnoses, tools/questionnaires used, and findings

Articles	Location	Study setting	Study type	*n*	Age	Inflammatory vulvar dermatosis	Tools/questionnaires	Tool to assess depression	Findings	Intervention
1	United Kingdom	Outpatient clinic	Prospective cohort	33	Mean: 50.5	Lichen sclerosus	FSFI, FSDS, HADS, RAS, Symptoms (visual analog scale for itching, burning, soreness), Pain Anxiety Symptoms Scale 20, Wound Management Questionnaire Revised, Vulvar Architecture Severity Scale.	HADS	Change in score of 3.5 (9.3-5.8) points overall following treatment	Fat grafting
2	New Zealand	Outpatient clinic	Prospective observational study	77	Media: 65 (18-85)	Lichen planus (17), lichen sclerosus (48), vulvar dermatitis NOS (12)	DLQI, HADS-A, HADS-D, FSDS, FSFI-S, FSFI-P	HADS	Worst depression scores were in women with LS (but not a significant difference). Rates of depression across patients were not different than those in the population at large (14% of women depressed in this study).	None
3	United States	Outpatient clinic	Survey	161	Mean: 39 (18-80)	Lichen planus or lichen sclerosus (*n* = 24, 15% of 161 patients with vulvar conditions)	FSFI, PHQ-9	PHQ-9	28 (20%) of women met criteria for moderate or severe depression. Could not assess % with LS or LP that had depression.	None
4	United Kingdom	Outpatient clinic	Survey	23	Media: 43 (19-77)	Eczema/lichen simplex chronicus (5), nonspecific or not documented (5), lichen sclerosus (3), lichen planus (3), pruritus (1), complex aphthosis (1), fragile fissuring vulval syndrome (1)	DLQI, HADS	HADS	Depression score was initially 8 or higher in 6 women (26%). Five had mild depression and 1 had severe depression. Highest depression scores were noted in patients with erosive LP and eczema. There was not a significant difference in changes in depression scores between initial and follow-up visits. Overall, low rates of depression in their patient population.	Interval between visits
5	Turkey	Outpatient clinic	Cross sectional study	60	Median:42.13 (vulvar dermatoses) (18-60)	Contact dermatitis (26), lichen simplex chronicus (*n* = 19), lichen planus (2), lichen sclerosus (*n* = 13)	Skindex-29 scale, WHO QOL BREF scale, HAM-A, HAM-D, Sociodemographic form,	HAM-D	Depression scores were not significantly different between patients with vulvar dermatoses, those with vulvar infections, and the control group. Average HAM-D for vulvar dermatoses group was 8.2. 50% of women with vulvar dermatoses had higher depression scores (HAM-D).	None
6	US	Outpatient clinic	Prospective cohort study	200	Mean: 38.4	Contact dermatitis (42), lichen simplex or lichen sclerosus (22)	CES-D, Cohen Perceived Stress scale, John Henry Scale	CES-D	43.5% of the patient population had previously diagnosed psychiatric conditions. Patients with contact dermatitis, LS and LSC were grouped into a group of other patients (104) of which 19 (18.3%) had a CES-D greater than 23.	None
7	Italy	Outpatient clinic	Prospective observational study	65	Median 53 for SCH and 60 for LS (27-60)	Vulvar squamous cell hyperplasia (44), lichen sclerosus (21)	CES-D, STAXI (STATE, TRAIT)	CES-D	No significant depressive status was diagnosed with CES-D in either group.	None
8	Poland	Outpatient clinic	Clinical trial	37	Mean: 59.98 (50-70)	Lichen sclerosus (37)	FSFI, BDI	BDI	Depressive symptoms in 48.65% of women; mild symptoms in 66.67% and moderate in 33.33%. After PDT, BDI scores improved (*P* = .003) though depressive symptoms remained unchanged.	PDT
9	United Kingdom	Peer support group; anonymous online survey	Survey	26	Unknown	Lichen sclerosus (22), lichen planus (4), eczema/psoriasis (2)	DLQI	Self-reported. No tool used	50% reported depression.	None
10	China	Outpatient clinic	Retrospective chart review	186	Mean: 45.6 (21-66)	Lichen sclerosus (51), lichen simplex chronicus (106), lichen planus (29)	Clinical efficacy, Self-Rating Depression scale, Self-Rating Anxiety scale	Self-reported. No tool used	27 patients had moderate or severe depression and or anxiety. Patients with depression had worse treatment outcomes	High-intensity focused ultrasound
11	United States	Outpatient clinic	Retrospective chart review	333	Median: 62.7 (23-90)	Lichen sclerosus	No tools	No tool utlized. Diagnosis of depression as co-morbidity	98 (31%) of patients with history of depression and/or anxiety	None
12	United States	Outpatient clinic	Retrospective chart review	33	Mean: 40: (18-45)	Lichen sclerosus	No tools	No tool utlized. Diagnosis of depression as comorbidity	21% had comorbid depression and/or anxiety	None
13	Portugal	Clinic	Retrospective chart review	228	Not available	Lichen sclerosus (228)	No tools	Self-reported. No tool used	Depression was an associated symptom with a higher risk of being symptomatic in patients with lichen sclerosus (RR of 2.55), but not statisticially significant.	None
14	Australia	Outpatient clinic	Survey	68	Median 58: (48-67)	Lichen sclerosus	VQLI	Self-reported. No tool used	Anxiety and or depression were seen in 13 (19%) of patients.	None

The first 8 articles are studies using validated depression instruments.

Study 6: Recurrent candidiasis, atrophic vaginitis, vulvar vestibulitis syndrome, desquamative inflammatory vaginitis, physiologic leukorrhea, bacterial vaginosis were also included in this study.

Study 9: *Respondents also included patients with vulvar conditions that were not inflammatory dermatoses: genital warts (2), vulvodynia (2), vulvar cancer (1)*.

*BDI*, Beck’s Depression Inventory; *CES-D*, Center for Epidemiologic Studies Depression scale; *DLQI*, dermatology life quality Index; *FSDS*, Female Sexual Distress scale; *FSFI*, female sexual function index; *HADS*, Hospital Anxiety and Depression Scale; *HAM-A* and *HAM-D*, Hamilton Anxiety Rating Scale and Hamilton Depression Rating Scale; *IEEF*, International Index of Erectile Function; *LP*, lichen planus; *LS*, lichen sclerosus; *PDT*, photodynamic therapy; *PHQ-9*, Patient Health Questionnaire; *PtGA*, patient’s global assessment; *RAS*, Relationship Assessment Scale; *Skindex*, Specific instrument for skin disease also assessing quality of life; *STAXI*, State-trait-anger Expression Inventory; *VAS*, visual analog scale; *VQLI*, Vulval Quality of Life Index; *WHO QOL*, World Health Organization Quality of Life BREF scale.

Overall, our review found that depression has been reported with each of these VIDs and the prevalence of depression among studied, ranged from 14% to 50%. One study in Italy found “no significant depressive status” in 21 patients with LS when using the Center for Epidemiologic Studies Depression Scale scale; however, Center for Epidemiologic Studies Depression Scale scores and frequency of administration of this scale was not included. All 8 studies with validated depression scales included LS and 3 studies on LS alone found depression in up to 48.65% of participants.

A recent systematic review of vulvar LS and LP, found both are associated with reduced quality of life and anxiety/depression may be associated.^4^ A case control study of a large research database found LS is significantly associated with depression and anxiety but was unable to separate vulvar from extragenital LS.^5^ Our review adds to these prior studies, summarizing the existing literature on depression in vulvar LS, LP, and lichen simplex chronicus. Limitations include the limited studies and study types, as most were surveys or retrospective chart reviews. Some studies combined depression and anxiety, and included other vulvovaginal conditions, not subdividing depression rates by VID.

Overall, we found depression and depressive symptoms are reported in patients with VID, with LS being the most common VID in the literature to report a depression assessment. However, there were few studies included and we believe the prevalence of depression found in this review, is likely underreported, due to the known impact these conditions have on quality of life outcomes.^1^ Due to these limitations, future prospective studies should consider screening for depression and the use of validated depression instruments in patients with VID, and if changes correlate with disease severity or treatment response.

## Conflicts of interest

None disclosed.
